# Relationships between cystatin C and creatinine‐based eGFR with low tongue pressure in Japanese rural community‐dwelling older adults

**DOI:** 10.1002/cre2.619

**Published:** 2022-06-24

**Authors:** Hiroshi Kusunoki, Yoko Hasegawa, Shotaro Tsuji, Yosuke Wada, Kayoko Tamaki, Koutatsu Nagai, Takara Mori, Ryota Matsuzawa, Hiromitsu Kishimoto, Hideo Shimizu, Ken Shinmura

**Affiliations:** ^1^ Division of General Medicine, Department of Internal Medicine Hyogo College of Medicine Nishinomiya Hyogo Japan; ^2^ Department of Internal Medicine Osaka Dental University Hirakata Osaka Japan; ^3^ Division of Comprehensive Prosthodontics Niigata University Graduate School of Medical and Dental Sciences Niigata Niigata Japan; ^4^ Amagasaki Medical COOP Honden Clinic Amagasaki Hyogo Japan; ^5^ Department of Orthopaedic Surgery Hyogo College of Medicine Nishinomiya Hyogo Japan; ^6^ School of Rehabilitation Hyogo University of Health Sciences Kobe Hyogo Japan; ^7^ Department of Dentistry and Oral Surgery Hyogo College of Medicine Nishinomiya Hyogo Japan

**Keywords:** chronic kidney disease (CKD), oral hypofunction, sarcopenia, tongue pressure

## Abstract

**Background:**

Sarcopenia is prevalent in patients with chronic kidney disease (CKD), which is defined as a low estimated glomerular filtration rate (eGFR). It has been reported that oral hypofunction characterized by decreased tongue pressure is related to sarcopenia. Although there are several previous reports regarding the association of renal dysfunction with oral hypofunction characterized by low tongue pressure, the association between tongue pressure and renal function is not fully understood.

**Methods:**

This cross‐sectional study included 68 men aged 79.0 ± 4.8 years and 145 women aged 77.3 ± 5.4 years from a rural area in Hyogo Prefecture, Japan. We examined the relationships between cystatin C‐based CKD (CKDcys), creatinine‐based CKD (CKDcre), ratio of cystatin C‐based GFR (eGFRcys) divided by creatinine‐based GFR (eGFRcre): eGFRcys/eGFRcre, and tongue pressure in community‐dwelling older adults.

**Results:**

Tongue pressure was significantly lower in participants with CKDcys than in those without CKDcys in men and women. However, there were no significant differences in tongue pressure with or without CKDcre. Tongue pressure was significantly lower in participants with eGFRcys/eGFRcre <1.0, than in those with eGFRcys/eGFRcre ≧ 1.0 in men. According to the receiver operating characteristic analysis, the optimal cut‐off value of tongue pressure for the presence of CKDcys was 36.6kPa, area under the curve (AUC) 0.74 (specificity 54.8%, sensitivity 84.6%) in men and 31.8kPa, AUC 0.65 (specificity 67.3%, sensitivity 60.5%) in women.

**Conclusions:**

CKDcys but not CKDcre is associated with low tongue pressure. In addition, a lower eGFRcys/eGFRcre ratio is a useful screening marker of low tongue pressure in community‐dwelling older adults.

## 1 INTRODUCTION

Oral health is essential for maintaining general health in the elderly population. In the Kashiwa study, Tanaka et al. (2018) reported that oral frailty is a risk factor for physical frailty, such as sarcopenia and mortality, in community‐dwelling elderly individuals. Recently, associations between sarcopenia and oral hypofunction have gained attention in geriatric medicine. The tongue plays a vital role in eating by compressing food against the palate and squeezing it into the pharynx during the oral preparatory phase of swallowing, after the food is pushed into the esophagus via pharyngeal wall contractions (Felton et al., 1985). Maximal voluntary tongue pressure is defined as the pressure to compress the balloon onto their palates as firmly as possible (Hayashi et al., 2002).

Previous studies have suggested that low masticatory ability is associated with weaker physical performance, sarcopenia, and mortality, whereas low tongue pressure is significantly associated with frailty in elderly individuals and reflects dysphagia (Tsuga et al., 2012; Yoshida et al., 2006). Tongue pressure is associated with swallowing function and closely associated with aspiration pneumonia. In the swallowing process, tongue pressure plays one of the most crucial roles, mixing food and saliva into a bolus and passing it into the pharynx (Kobuchi et al., 2020). Dysphagia is associated with reduced swallowing and is caused by several factors, such as decreased tongue pressure and decreased elaboration of tongue movements. Decreased tongue pressure is associated with dysphagia due to sarcopenia in bedridden elderly patients (Maeda & Akagi, 2015). As sarcopenia is involved in the onset of dysphagia (Wakabayashi, 2014; Wakabayashi & Sakuma, 2014) the relationship between sarcopenia and dysphagia has attracted attention. Tongue strength declines in patients with sarcopenia (Machida et al., 2017).

It was reported that there is a high prevalence of frailty in patients with chronic kidney disease (CKD) and a significant association between frailty and motor skills, affecting swallowing in patients with CKD (Kosaka et al., 2020). Kamijo et al. (2018) reported that in peritoneal dialysis patients, low tongue pressure is a risk factor for sarcopenia. Another study reported that impaired oral health evaluated using the Revised Oral Assessment Guide (ROAG) was closely associated with CKD in postacute inpatients, and estimated glomerular filtration rate (eGFR) was independently associated with the ROAG score after adjusting for possible confounders (Shiraishi et al., 2021). There are several previous reports stating that oral hypofunction, characterized by low tongue pressure, is associated with overt renal dysfunction, although this phenomenon is not fully understood. Low tongue pressure is associated with renal function even in elderly individuals without severe renal impairment.

Renal function is usually evaluated using the eGFR. The use of cystatin C (CysC) strengthens the association between the eGFR and the risk of death and end‐stage renal disease (ESRD). CysC‐based eGFR values (eGFRcys) are more reliable than creatinine‐based eGFR values (eGFRcre) for determining long‐term prognosis (Shlipak et al., 2013). The eGFRcre is determined using both renal function and muscle mass. A previous study reported an association between eGFRcys reduction and an increase in the prevalence and incidence of frailty, which was not observed with eGFRcre reduction (Dalrymple et al., 2013). Furthermore, eGFRcys is related to a higher risk of sarcopenia than eGFRcre because eGFRcys is not affected by low muscle mass or quality (Baxmann et al., 2008). A low eGFRcys (CKDcys), but not a low eGFRcre (CKDcre), is reported to be independently related to osteoporotic fracture occurrence in postmenopausal women. Moreover, the eGFRcys/eGFRcre ratio was independently related to osteoporotic fracture in this study and was correlated to physical function. The eGFRcys/eGFRcre ratio may be a clinically useful parameter for muscle mass loss assessment (Kurajoh et al., 2019). Several studies have reported a correlation between the occurrences of sarcopenia and osteoporosis (Go et al., 2013; Miyakoshi et al., 2013). We speculate that eGFRcys is superior to eGFRcre in evaluating muscle mass and physical function and is more strongly associated with sarcopenia. Therefore, we recently reported a relationship between cystatin C‐based eGFR (eGFRcys) was associated with sarcopenia. But creatinine‐based eGFR (eGFRcre) was not associated with sarcopenia. The presence of low eGFRcys (CKDcys) and low eGFRcys/eGFRcre ratio (<1.0), but not with low eGFRcre (CKDcre) were associated with systemic sarcopenia (Kusunoki, Tsuji, et al., 2021).

Oral frailty has been shown to be a risk factor for physical frailty, sarcopenia, and disability in community‐dwelling elderly individuals (Tanaka et al., 2018). Tongue pressure is positively correlated with physical performance and grip strength and thus is used for detecting subclinical dysphagia (K. C. Chen et al., 2021). Tongue pressure may also be a sensitive marker of systemic organ failure such as subclinical CKD.

We hypothesized that a similar phenomenon observed in systemic sarcopenia in our previous article (Kusunoki, Tsuji, et al., 2021) occurs in oral hypofunction characterized by low tongue pressure. The aim of this study was to clarify whether low tongue pressure was associated with renal function and explain the differences between CKDcre and CKDcys.　As previously described, a dissociation between eGFRcre and eGFRcys is associated with various organ failures (Shlipak et al., 2013). Hence, we evaluated whether eGFRcys/eGFRcre ratio <1.0 was associated with tongue pressure.

## 2 MATERIALS

### 2.1 Study participants

This cross‐sectional study was called the Frail Elderly in the Sasayama‐Tamba Area (FESTA) study. The study population comprised individuals aged ≥65 years. Healthy community‐dwelling elderly individuals from the Sasayama Tamba area, a rural area in Hyogo Prefecture, Japan, were recruited between 2015 and 2019. Measurement of body composition and blood sample analysis were performed as described previously (Kusunoki, Tsuji, et al., 2021; Kusunoki, Tabara, et al., 2021). Body composition was evaluated via bioelectrical impedance analysis (BIA) using an InBody 770 device (InBody Japan Inc.). The skeletal muscle mass index (SMI) was calculated as skeletal muscle mass (SMM)/height‐squared (kg/m^2^). Handgrip strength was evaluated as described previously (L. K. Chen et al., 2020; Kusunoki, Tsuji, et al., 2021; Kusunoki, Tabara, et al., 2021).

All procedures performed in studies involving human participants were in accordance with the ethical standards of the institutional and/or national research committee where the studies were conducted (IRB approval number Rinhi 0342 at Hyogo College of Medicine) and with the 1964 Helsinki declaration and its later amendments or comparable ethical standards.

### 2.2 Evaluation of physical function

For the assessment of gait speed, we asked participants to cover a 12 m walkway at their usual speed, during which the time to walk 10 m was assessed. We measured maximum grip strength using a grip strength tester (GRIP‐A; Takei Ltd., Niigata, Japan) (Nagai et al., 2020). Knee extension strength (Nm) was measured in the dominant leg during isometric contraction of the knee extensor in the sitting position, with the knee position maintained at 60, using a hand‐held dynamometer (Sakai Medical. Co., Ltd., Tokyo, Japan) (Kusunoki et al., 2018).

### 2.3 Evaluation of oral function

The participants sat in reclinable nursing chairs and underwent oral examinations. We assessed the remaining teeth, occlusal force, and tongue pressure. For the occlusal force, we measured the maximum occlusal force of the first left and right molars using an occlusal force meter (Occlusal Force‐Meter GM10; NAGANO KEIKI, Tokyo, Japan) and evaluated the sum of the two.

For tongue pressure, we measured the maximum tongue pressure twice using a JMS Tongue Pressure Measuring Device (JMS Co. Ltd., Hiroshima, Japan) and took the highest value (Hasegawa et al., 2021). We defined tongue pressure <30 kPa as low tongue pressure according to the criteria of the Japan Dental Association (Minakuchi et al., 2016).

### 2.4 Categorization of CKD

CKD was defined and classified according to the Kidney Disease: Improving Global Outcomes (KDIGO) criteria (National Kidney Foundation, 2002). We calculated the eGFRcre and eGFRcys using equations from the Japanese Society of Nephrology (Horio et al., 2013; Matsuo et al., 2009). Low eGFRcre (CKDcre) was defined as an eGFRcre <60 ml/min/1.73 m^2^, and low eGFRcys (CKDcys) as an eGFR <60 ml/min/1.73 m^2^. The SMI was calculated as SMM/height‐squared (kg/m^2^).

### 2.5 Statistical analysis

The results are expressed as the mean±standard deviation (SD) or percentages. For intergroup comparisons, a Student's *t* test was used for data analysis. Pearson's product moment correlation coefficient was used to assess the associations between tongue pressure and age, height, body weight, body mass index (BMI), renal function parameters, muscle volume and strength parameters, usual and maximal gait speed, number of teeth, and occlusal force.

Categorical variables are expressed as absolute (*n*) and relative frequency (%) and were analyzed using Fisher's exact test. Univariate and multivariate logistic regression analyses were performed to calculate the odds ratio and 95% confidence interval (CI). A receiver operating characteristic curve (ROC) analysis was performed to confirm the diagnostic efficacy of tongue pressure for CKDcre and CKDcys. The area under the curve (AUC) was then calculated. JMP 13.1 software was used for data analysis. Statistical significance was set at *p* < .05.

## 3 RESULTS

The baseline characteristics, indices of body composition, and physical performance of the participants are presented in Table 1. The study included 68 men aged 65–92 years and 145 women aged 65–93 years. Grip power, knee extension muscle strength, SMM, and SMI were higher in men than in women (*p* < .001) (Table 1). The average eGFRcre was 63.3 (men: 64.2, women: 62.8). The average eGFRcys was 67.0 (men: 64.6, women: 68.2). There were no end‐stage renal disease (ESRD) patients with renal replacement therapy, and renal function was preserved (eGFRcre > 50) in most participants.

**Table 1:** Clinical characteristics, muscle volume, and physical performance of the participants

	Total (*n* = 213)	Men (*n* = 68)	Women (*n* = 145)	*p*
Age (year)	77.8 ± 5.3	79.0 ± 4.8	77.3 ± 5.4	.032
Height (cm)	154.4 ± 8.2	163.7 ± 5.5	150.1 ± 5.1	<.001
Body weight (kg)	54.2 ± 9.5	62.2 ± 9.6	50.5 ± 9.5	<.001
Body mass index	22.6 ± 2.9	23.2 ± 2.9	22.4 ± 2.8	.064
Cre (mg/dl)	0.80 ± 0.47	0.90 ± 0.18	0.75 ± 0.55	.026
CysC (mg/L)	1.03 ± 0.36	1.10 ± 0.26	0.99 ± 0.40	.054
Cre/CysC	0.77 ± 0.12	0.84 ± 0.11	0.74 ± 0.11	<.001
eGFRcre (ml/min/1.73 m^2^)	63.3 ± 13.3	64.2 ± 11.7	62.8 ± 13.9	.485
eGFRcys (ml/min/1.73 m^2^)	67.0 ± 15.9	64.6 ± 16.3	68.2 ± 15.6	.123
eGFRcys/eGFRcre	1.06 ± 0.16	1.00 ± 0.16	1.09 ± 0.15	<.001
CKDcre, *n* (%)	75 (35.2)	22 (32.4)	53 (36.6)	.645
CKDcys, *n* (%)	64 (30.0)	26 (38.2)	38 (26.2)	.080
eGFRcys/eGFRcre <1.0, *n* (%)	78 (36.6)	36 (52.9)	42 (29.0)	<.001
Skeletal muscle mass index (SMI)	6.33 ± 0.90	7.28 ± 0.72	5.89 ± 0.57	<.001
Skeletal muscle mass (SMM) (kg)	15.8 ± 3.6	19.6 ± 2.7	13.3 ± 1.7	<.001
Grip power (kg)	25.7 ± 6.6	33.0 ± 5.2	22.3 ± 3.8	<.001
Knee extension muscle strength (Nm)	303.1 ± 98.7	380.3 ± 101.5	266.9 ± 73.6	<.001
Usual gait speed (m/s)	1.39 ± 0.22	1.36 ± 0.20	1.41 ± 0.22	.150
Maximal gait speed (m/s)	1.78 ± 0.28	1.82 ± 0.30	1.76 ± 0.26	.109
Tongue pressure (kPa)	33.3 ± 8.4	33.7 ± 9.1	33.1 ± 8.1	.644
Number of teeth, *n*	19.1 ± 9.0	17.9 ± 9.9	19.6 ± 8.6	.190
Number of low tongue pressure, *n* (%)	74(34.7)	23(33.8)	51(35.1)	.847
Occlusal force (kgf)	44.8 ± 34.7	46.2 ± 40.1	44.1 ± 32.0	.693

Among the 213 participants, 74 (23 men, and 51 women) had low tongue pressure. In total, 67 (22 men, 45 women) participants had low eGFRcre (CKDcre), and 64 (26 men, 38 women) had low eGFRcys (CKDcys). In total, 78 participants (36 men, 42 women) had a low eGFRcys/eGFRcre ratio (<1.0).

Table 2 reports the correlations between tongue pressure and age, height, body weight, BMI, the parameters of muscle volume‐based BIA (such as SMI and SMM), muscle strength and physical function parameters (grip power, knee extension muscle strength, usual gait speed, and maximal gait speed), number of teeth, and occlusal force.

**Table 2:** Correlations between tongue pressure and the parameters of clinical characteristics, muscle volume, and physical performance

	Men (*n* = 68)		Women (*n* = 145)	
	*r*	*p*	*r*	*p*
Age (year)	−.66	<.001	−.31	<.001
Height (cm)	.26	.029	.06	.474
Body weight (kg)	.24	.050	.18	.030
Body mass index	.14	.248	.16	.058
Cre (mg/dl)	−.20	.110	−.08	.330
CysC (mg/L)	−.40	<.001	−.13	.122
Cre/CysC	.35	.003	.04	.664
eGFRcre (ml/min/1.73 m^2^)	.23	.062	.13	.126
eGFRcys (ml/min/1.73 m^2^)	.38	.002	.19	.025
eGFRcys/eGFRcre	.39	.001	.09	.281
Skeletal muscle mass index (SMI)	.27	.024	.21	.012
Skeletal muscle mass (SMM) (kg)	.33	.006	.19	.019
Grip power (kg)	.33	.006	.28	<.001
Knee extension muscle strength (Nm)	.38	.001	.32	<.001
Usual gait speed (m/s)	.20	.108	.21	.011
Maximal gait speed (m/s)	.33	.006	.25	.002
Number of teeth, *n*	.12	.340	−.09	.276
Occlusal force (kgf)	.26	.031	.08	.370

Consistent with previous reports, age, SMI, SMM, grip power, and knee extension muscle strength were significantly correlated with tongue pressure in men and women. Interestingly, eGFRcys, but not eGFRcre, were positively correlated with tongue pressure. Cre/CysC and eGFRcys/eGFRcre were positively correlated with tongue pressure in men but not in women. Maximal gait speed was positively correlated with tongue pressure in men and women. Usual gait speed was positively correlated with tongue pressure in women but not in men. The number of teeth was not correlated with tongue pressure. However, the occlusal force was positively correlated with tongue pressure only in men.

The participants were divided according to the presence or absence of CKDcre (Table 3A), CKDcys (Table 3B), and eGFRcys/eGFRcre < 1.0 (Table 3C). The average of each parameter is shown. There was no significant difference in age, SMI, SMM, grip power, knee extension muscle strength, maximal gait speed, number of teeth, and tongue pressure between participants with or without CKDcre in men and women (Table 3A). There was no significant difference in age, SMI, SMM, number of teeth, and occlusal force between participants with or without CKDcys in men and women. However, tongue pressure was significantly higher in participants without CKDcys than in those with CKDcys. Similarly, there was no significant difference in age, SMI, SMM, and number of teeth between participants with eGFRcys/eGFRcre < 1.0 and eGFRcys/eGFRcre ≧ 1.0 in men and women. Tongue pressure was significantly higher in men with eGFRcys/eGFRcre < 1.0 than in those with eGFRcys/eGFRcre ≧ 1.0. We showed by univariate logistic regression analysis that eGFRcys, but not eGFRcre, was associated with low tongue pressure (<30kPa) in men and women (Tables 4A and B) (*p* < .05). Moreover, eGFRcys/eGFRcre < 1.0 was associated with low tongue pressure in men and women (Tables 4A and B). In addition, eGFRcys, but not eGFRcys/eGFRcre and eGFRcre, was associated with low tongue pressure in women (*p* < .05) (Table 4B).

**Table 3A:** Characteristics of participants with and without CKDcre

	Men (*n* = 68)			Women (*n* = 145)		
	CKDcre(−) (*n* = 46)	CKDcre(+) (*n* = 22)	*p*	CKDcre(−) (*n* = 92)	CKDcre(+) (*n* = 53)	*p*
Age (year old)	79.0 ± 5.0	79.0 ± 4.3	.999	77.4 ± 4.9	77.0 ± 6.2	.663
Height (cm)	163.0 ± 5.9	165.1 ± 4.4	.152	150.0 ± 5.3	150.4 ± 4.9	.674
Body weight (kg)	60.8 ± 8.7	65.1 ± 10.9	.083	50.2 ± 6.9	51.0 ± 6.7	.465
Body mass index	22.8 ± 2.4	23.9 ± 3.8	.166	22.3 ± 2.9	22.6 ± 2.7	.594
Cre (mg/dl)	0.81 ± 0.07	1.11 ± 0.18	<.001	0.62 ± 0.06	0.98 ± 0.86	<.001
CysC (mg/L)	0.99 ± 0.18	1.32 ± 0.28	<.001	0.86 ± 0.10	1.23 ± 0.59	<.001
Cre/CysC	0.83 ± 0.12	0.85 ± 0.10	.402	0.72 ± 0.09	0.77 ± 0.13	.003
eGFRcre (ml/min/1.73 m^2^)	70.7 ± 6.7	50.6 ± 7.6	<.001	70.8 ± 8.3	48.9 ± 10.3	<.001
eGFRcys (ml/min/1.73 m^2^)	70.8 ± 14.0	51.5 ± 12.6	<.001	75.7 ± 10.4	55.0 ± 14.4	<.001
eGFRcys/eGFRcre	1.00 ± 0.16	1.01 ± 0.15	.754	1.07 ± 0.15	1.13 ± 0.16	.052
Grip power (kg)	32.3 ± 5.0	34.5 ± 5.3	.087	22.6 ± 3.9	21.8 ± 3.5	.270
Knee extension muscle strength (Nm)	381.2 ± 104.7	378.4 ± 96.7.	.916	266.8 ± 70.7	266.9 ± 79.2	.996
Skeletal muscle mass (SMM) (kg)	19.1 ± 2.7	20.5 ± 2.6	.059	13.3 ± 1.8	13.3 ± 1.7	.942
Skeletal muscle mass index (SMI)	7.17 ± 0.64	7.50 ± 0.83	.075	5.89 ± 0.56	5.88 ± 0.59	.877
Usual gait speed (m/s)	1.39 ± 0.17	1.29 ± 0.25	.044	1.42 ± 0.23	1.38 ± 0.21	.373
Maximal gait speed (m/s)	1.85 ± 0.26	1.77 ± 0.38	.229	1.79 ± 0.27	1.71 ± 0.24	.079
Number of teeth, *n*	16.9 ± 9.5	19.9 ± 10.5	.241	19.0 ± 8.4	20.7 ± 8.9	.238
Tongue pressure (kPa)	34.0 ± 8.9	33.1 ± 9.5	.711	34.0 ± 7.7	31.6 ± 8.5	.083
Number of low tongue pressure, *n* (%)	15 (32.6)	8 (36.4)	.789	27 (29.3)	24 (45.3)	.071
Occlusal force (kgf)	46.6 ± 43.1	45.3 ± 34.0	.902	43.2 ± 32.5	45.8 ± 31.4	.632

**Table 3B:** Characteristics of participants with and without CKDcys

	Men (*n* = 68)			Women (*n* = 145)		
	CKDcys(−) (*n* = 42)	CKDcys(+) (*n* = 26)	*p*	CKDcys(−) (*n* = 107)	CKDcys(+) (*n* = 38)	*p*
Age (year old)	78.3 ± 4.5	80.0 ± 5.2	.158	77.1 ± 4.9	77.9 ± 6.7	.429
Height (cm)	163.8 ± 5.8	163.5 ± 5.2	.806	150.3 ± 5.2	149.7 ± 5.1	.574
Body weight (kg)	61.7 ± 9.7	63.0 ± 9.4	.601	49.7 ± 6.6	52.7 ± 7.1	.021
Body mass index	22.9 ± 2.9	23.5 ± 3.1	.412	22.0 ± 2.7	23.5 ± 2.9	.005
Cre (mg/dl)	0.82 ± 0.09	1.05 ± 0.20	<.001	0.64 ± 0.09	1.05 ± 1.02	<.001
CysC (mg/L)	0.94 ± 0.11	1.36 ± 0.23	<.001	0.86 ± 0.09	1.36 ± 0.65	<.001
Cre/CysC	0.88 ± 0.11	0.78 ± 0.09	<.001	0.75 ± 0.09	0.72 ± 0.14	.189
eGFRcre (ml/min/1.73 m^2^)	70.3 ± 7.9	54.3 ± 10.1	<.001	68.1 ± 10.0	48.0 ± 12.9	<.001
eGFRcys (ml/min/1.73 m^2^)	74.4 ± 11.2	48.6 ± 8.6	<.001	75.3 ± 9.7	48.1 ± 11.1	<.001
eGFRcys/eGFRcre	1.06 ± 0.15	0.91 ± 0.12	<.001	1.12 ± 0.15	1.02 ± 0.14	<.001
Grip power (kg)	32.6 ± 4.9	33.6 ± 5.6	.442	22.8 ± 3.7	20.9 ± 3.8	.006
Knee extension muscle strength (Nm)	394.0 ± 93.0	358.2 ± 112.2	.159	274.3 ± 73.0	245.9 ± 72.0	.040
Skeletal muscle mass index (SMI)	7.24 ± 0.65	7.34 ± 0.82	.597	5.86 ± 0.58	5.98 ± 0.53	.252
Skeletal muscle mass (SMM) (kg)	19.5 ± 2.7	19.7 ± 2.7	.840	13.3 ± 1.8	13.4 ± 1.6	.619
Usual gait speed (m/s)	1.41 ± 0.17	1.27 ± 0.22	.006	1.43 ± 0.21	1.33 ± 0.22	.012
Maximal gait speed (m/s)	1.88 ± 0.24	1.73 ± 0.37	.037	1.81 ± 0.26	1.63 ± 0.22	<.001
Number of teeth, *n*	18.3 ± 9.1	17.2 ± 11.2	.678	19.9 ± 8.4	18.7 ± 9.0	.455
Tongue pressure (kPa)	36.6 ± 8.5	29.0 ± 8.0	<.001	34.1 ± 8.2	30.3 ± 7.0	.012
Number of low tongue pressure, *n* (%)	10 (23.8)	13 (50.0)	.036	31 (29.0)	20 (52.6)	.011
Occlusal force (kgf)	50.1 ± 41.7	39.9 ± 37.3	.310	46.6 ± 33.7	37.3 ± 25.8	.124

**Table 3C:** Characteristics of participants with and without eGFRcys/eGFRcr < 1.0

	Men (*n* = 68)			Women (*n* = 145)		
	eGFRcys/eGFRcr ≧ 1.0 (*n* = 32)	eGFRcys/eGFRcr＜1.0 (*n* = 36)	*p*	eGFRcys/eGFRcr ≧ 1.0 (*n* = 103)	eGFRcys/eGFRcr＜1.0 (*n* = 42)	*p*
Age (year old)	77.8 ± 4.2	79.9 ± 5.1	.070	77.0 ± 5.3	78.1 ± 5.5	.243
Height (cm)	164.4 ± 5.4	163.0 ± 5.6	.280	150.3 ± 5.1	149.7 ± 5.4	.466
Body weight (kg)	62.7 ± 10.0	61.8 ± 9.3	.690	50.0 ± 6.3	51.7 ± 7.9	.168
Body mass index	23.1 ± 3.2	23.2 ± 2.8	.952	22.1 ± 2.6	23.1 ± 3.1	.070
Cre (mg/dl)	0.90 ± 0.18	0.91 ± 0.19	.973	0.77 ± 0.64	0.69 ± 0.20	.419
CysC (mg/L)	0.98 ± 0.22	1.21 ± 0.26	<.001	0.96 ± 0.44	1.08 ± 0.28	.087
Cre/CysC	0.93 ± 0.08	0.75 ± 0.06	<.001	0.78 ± 0.10	0.64 ± 0.03	<.001
eGFRcre (ml/min/1.73 m^2^)	64.3 ± 11.4	64.1 ± 12.2	.930	61.8 ± 13.5	65.4 ± 14.9	.153
eGFRcys (ml/min/1.73 m^2^)	73.3 ± 15.8	56.8 ± 12.3	<.001	71.3 ± 15.3	60.3 ± 13.7	<.001
eGFRcys/eGFRcre	1.14 ± 0.11	0.89 ± 0.08	<.001	1.16 ± 0.12	0.92 ± 0.05	<.001
Grip power (kg)	33.7 ± 5.6	32.4 ± 4.7	.314	22.7 ± 3.7	21.2 ± 3.8	.030
Knee extension muscle strength (Nm)	391.4 ± 79.1	370.5 ± 118.2	.401	279.3 ± 74.8	236.3 ± 61.3	.001
Skeletal muscle mass (SMM) (kg)	20.2 ± 2.9	19.0 ± 2.5	.074	13.3 ± 1.7	13.3 ± 2.0	.816
Skeletal muscle mass index (SMI)	7.44 ± 0.76	7.14 ± 0.66	.081	5.88 ± 0.53	5.90 ± 0.65	.904
Usual gait speed (m/s)	1.38 ± 0.23	1.34 ± 0.18	.363	1.41 ± 0.21	1.38 ± 0.25	.465
Maximal gait speed (m/s)	1.86 ± 0.30	1.79 ± 0.30	.333	1.79 ± 0.27	1.69 ± 0.24	.060
Number of teeth, *n*	18.6 ± 8.8	17.2 ± 10.8	.571	19.8 ± 8.5	19.2 ± 8.8	.695
Tongue pressure (kPa)	36.8 ± 8.8	30.9 ± 8.5	.007	33.4 ± 8.4	32.3 ± 7.2	.421
Number of low tongue pressure, *n* (%)	6 (18.8)	17 (47.2)	.020	37 (35.9)	14 (33.3)	.849
Occlusal force (kgf)	49.5 ± 39.6	43.2 ± 40.9	.521	45.7 ± 32.5	40.5 ± 30.7	.382

**Table 4:** Univariate logistic regression analysis of factors associated with low tongue pressure in men (A) and women (B)

A. Men			
	OR	95% CI	*p*
CKDcre	1.18	0.41‐3.43	.760
CKDcys	3.20	1.12‐9.11	.027
eGFRcys/eGFRcre < 1.0	3.88	1.29‐11.68	.012

B. Women			
	OR	95% CI	*p*
CKDcre	1.99	0.99‐4.02	.054
CKDcys	2.72	1.27‐5.83	.010
eGFRcys/eGFRcre < 1.0	1.12	0.53‐2.39	.767

Abbreviations: CI, confidence interval; OR, odds ratio.

**Figure 1:**
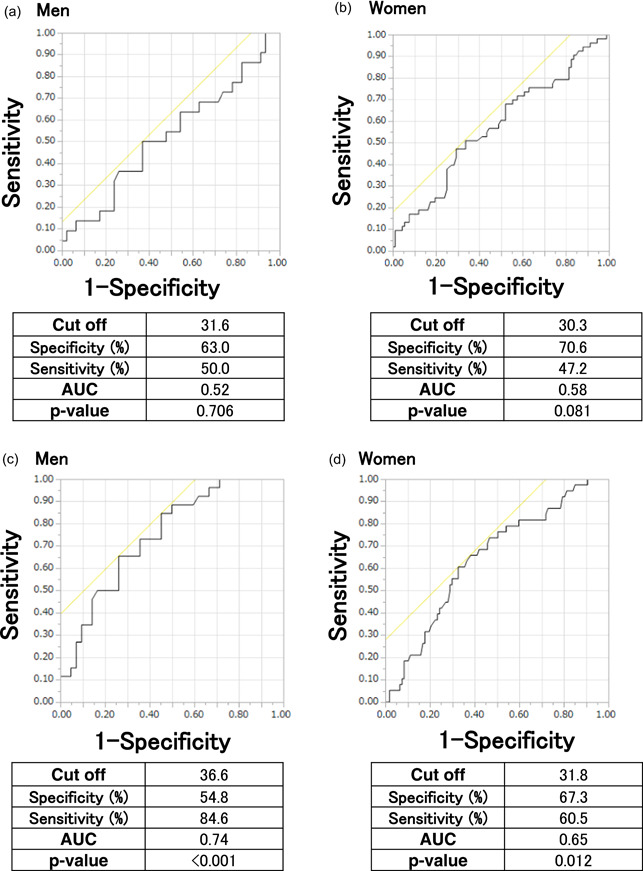
Receiver operating characteristic curves for tongue pressure and CKDcre in men (a) and women (b). Receiver operating characteristic curves for tongue pressure and CKDcys in men (c) and women (d). AUC, area under the curve.

Figure 1a,b shows the ROC curves of CKDcre for identifying low tongue pressure in men (A) and women (B). There was no significant association between CKDcre and low tongue pressure. Figure 1c,d shows the ROC curves of CKDcys for identifying low tongue pressure in men (C) and women (D). There was a significant association between CKDcys and low tongue pressure. The AUC of eGFRcys was 0.74 in men and 0.65 in women. The cut‐off value was 36.6 kPa in men and 31.8 kPa in women.

## 4 DISCUSSION

The current study shows the relationships between cystatin C and creatinine‐based eGFR in Japanese rural community‐dwelling older adults with oral dysfunction characterized by low tongue pressure. Consistent with previous reports, the current study shows that tongue pressure is negatively correlated with age and positively correlated with indices of sarcopenia diagnosis, such as muscle volume and grip strength (Kaji et al., 2019; Kobuchi et al., 2020; Maeda & Akagi, 2015; Utanohara et al., 2008). Furthermore, this study shows that the maximal gait speed is positively correlated with tongue pressure.

Although there was no significant difference in tongue pressure between the participants with and without CKDcre, tongue pressure was significantly weaker in participants with CKDcys than in those without CKDcys. Interestingly, there were no significant differences between participants with and without CKDcys regarding age, SMM, SMI, occlusal force, and number of teeth.

Yamada et al. (2017) previously reported that muscle quality is a more valuable measure than muscle quantity to indicate muscle function. While skeletal muscle contains peri‐muscular adipose tissues or connective tissues, the tongue is a pure muscular organ composed of intrinsic and extrinsic muscles. Systemic muscle quality may be affected by subclinical and potential renal injuries. Tongue pressure may reflect systemic muscle quality more keenly than skeletal muscle mass as determined via BIA.

eGFRcys is less influenced by low muscle quantity or quality than is eGFRcre. Tongue pressure can be a more sensitive marker than muscle quantity because tongue pressure may reflect subclinical organ damage, such as CKDcys. Thus, CKDcys could explain why tongue pressure was significantly weaker in participants with CKDcys than in those without CKDcys, while there was no significant difference between participants with and without CKDcys in muscle quantity.

Univariate logistic regression analysis showed that eGFRcys but not eGFRcre, were associated with low tongue pressure (<30kPa) both in men and women.　In our previous study, multivariate logistic regression analysis showed that an eGFRcys/eGFRcre ratio < 1.0 was associated with sarcopenia in men but not in women (Kusunoki, Tsuji, et al., 2021). Similarly, univariate logistic regression analysis showed that eGFRcys/eGFRcre < 1.0 was associated with low tongue pressure (<30kPa) in men but not women (Table 4). Generally, total muscle volume is higher in men than in women. There is a lower change in muscle volume in CysC than in Cre. Therefore, we expected the change in eGFRcys/eGFRcre due to the decrease in muscle mass to be more prominent in men than in women. The difference in body composition between sexes and the discrepancy in the influence on eGFRcys/eGFRcre in association with the change in muscle volume between men and women may explain this difference.

There is a significant association between CKDcys and low tongue pressure in the ROC analysis. The cut‐off tongue pressure value was 36.6 kPa in men and 31.8 kPa in women. This cut‐off value is higher than the 30 kPa cut‐off value for low tongue pressure determined by the Japan Dental Association (Minakuchi et al., 2016).

In previous reports, 853 participants without a history of dysphagia and maintained occlusal contact in the premolar and molar regions with their own teeth were divided into six age groups: 20–29, 30–39, 40–49, 50–59, 60–69, and 70–79 years. The tongue pressure average was more than 30 kPa in all groups (Utanohara et al., 2008). They therefore reasoned that most healthy elderly individuals with maintained occlusal contact with their own teeth have a tongue pressure more than 30 kPa, and thus the cut‐off value of low tongue pressure was determined to be <30 kPa by the Japan Dental Association (Kusunoki, Tsuji, et al., 2021).

The cut‐off values of tongue pressure for more evident organ damage, such as physical frailty, sarcopenia, and aspiration pneumonia, are lower than 30 kPa. The Kashiwa study showed that tongue pressure <27.4 kPa in men and <26.5 kPa in women was significantly associated with physical frailty and sarcopenia (Tanaka et al., 2018). Nakamori et al. reported that tongue pressure is a sensitive indicator for predicting the occurrence of pneumonia in acute stroke patients. They performed a ROC analysis to establish the ability of tongue pressure to predict the modified Mann Assessment of Swallowing Ability (MASA) score <95, which suggests swallowing dysfunction. The optical cutoff point for tongue pressure was 21.6 kPa. They showed that lower tongue pressure (<21.6 kPa) was an independent risk factor for pneumonia (Nakamori et al., 2016). In studies using the JMS device, Japanese elderly subjects with frailty showed a maximum tongue pressure of 18.0 ± 12.0 kPa (Tsuga et al., 2012; Yoshida et al., 2006). In patients with spinal and bulbar muscular atrophy, the maximum tongue pressure in patients with severe dysphagia was approximately <20 kPa (Mano et al., 2014).

CKD is a risk factor for cardiovascular disease and is associated with increased all‐cause mortality. As mentioned in a previous study (Kusunoki, Tsuji, et al., 2021) CysC may be influenced by mild chronic inflammation and oxidative stress (Xu et al., 2015). Thus, eGFRcys may be more sensitive to mild inflammatory and oxidative changes in sarcopenia than is eGFRcre. As CKDcys may be a presymptomatic state of evident organ damage, the cut‐off values of tongue pressure for CKDcys were higher than those for more severe diseases, physical frailty, sarcopenia, and pneumonia.

It has been reported that tooth loss affects tongue pressure in healthy elderly subjects (Kikutani et al., 2009). However, there were no significant differences in the number of teeth between participants with or without CKDcys and eGFRcys/eGFRcre < 1.0 in both men and women. Although the number of remaining teeth may affect tongue pressure, the contribution to the healthy oral function of tongue pressure is more significant than that of the remaining teeth.

Our study has limitations we must consider. First, this was a cross‐sectional study, and we cannot determine any cause‐and‐effect relationship. A follow‐up prospective study is needed to assess any causal associations between CKD and low tongue pressure. Second, most participants volunteered for this study. Therefore, they are likely to have been healthier. The study population was also likely to have lower rates of oral hypofunction than those in the general population. This lower rate could account for the inconsistency between our results and previous studies. Finally, we recruited a small sample size. This limits the reliability and validity of the tests.

In conclusion, in our study, CKDcys, but not CKDcre, was associated with low tongue pressure. In addition, a lower eGFRcys/eGFRcre ratio was a useful screening marker for low tongue pressure in community‐dwelling older adults. As observed in patients with systemic sarcopenia, a dissociation between eGFRcre and eGFRcys is associated with low tongue pressure. Thus, tongue pressure may be a sensitive marker of subclinical renal impairment. We observed and reported that even in elderly individuals without severe renal impairment, a similar phenomenon in systemic sarcopenia occurs in oral hypofunction, characterized by low tongue pressure.

## AUTHOR CONTRIBUTIONS

All authors were involved in data collection and data analysis. Hiroshi Kusunoki and Yoko Hasegawa were involved in study design and data interpretation. Hiromitsu Kishimoto, Hideo Shimizu, and Ken Shinmura were involved in data analysis and critical review. All authors critically revised the manuscript, commented on drafts of the manuscript, approved the manuscript to be published, and agreed to be accountable for all aspects of the work, ensuring that questions related to the accuracy or integrity of any part of the work are appropriately investigated and resolved.

## CONFLICT OF INTEREST

The author declares no conflict of interest.

## ETHICS STATEMENT

All procedures performed in studies involving human participants were in accordance with the ethical standards of the institutional and/or national research committee at which the studies were conducted (IRB approval number Rinhi 0342 at Hyogo College of Medicine) and with the 1964 Helsinki declaration and its later amendments or comparable ethical standards. Informed consent was obtained from all individual participants included in the study.

## Data Availability

The data that support the findings of this study are available from the corresponding author upon reasonable request.
